# Testing theories of policy growth: public demands, interest group politics, electoral competition, and institutional fragmentation

**DOI:** 10.1080/13501763.2024.2317358

**Published:** 2024-02-27

**Authors:** Xavier Fernández-i-Marín, Markus Hinterleitner, Christoph Knill, Yves Steinebach

**Affiliations:** a Department of Political Science, University of Barcelona, Barcelona, Spain; b Swiss Graduate School of Public Administration, University of Lausanne, Chavannes-près-Renens, Switzerland; c Geschwister Scholl Institute for Political Science, LMU Munich, Munich, Germany; d Department of Political Science, University of Oslo, Oslo, Norway

**Keywords:** Policy growth, democracy, interest groups, policy state, environmental policy

## Abstract

Policy growth is a ubiquitous feature of modern democracies that has attracted increased attention in political science and beyond. However, the literature is characterised by considerable disagreement on why policy growth occurs. Existing explanations centre on the influence of (1) public demands, (2) interest group politics, (3) electoral competition, and (4) institutional fragmentation. While all four explanations are plausible, there are no studies that assess their relative explanatory power within a single empirical analysis. This article provides such an analysis by examining the drivers of policy growth in 21 OECD countries from 1976 to 2020 in the area of environmental policy. We identify strong ties between organised interests and the government as the primary driver of policy growth. Public demands and institutional fragmentation are relevant but comparatively less important factors, while the intensity of electoral competition has no influence on policy growth. These findings have important implications for our understanding of the functioning of democracy in the long run.

## Introduction

A diverse and growing body of political science literature has identified policy growth as a ubiquitous feature of modern democracies. Concepts and terms such as ‘continuous rule growth,’ ‘policy layering,’ or ‘policy accumulation’ all seek to capture the observation that, over time, democratic governments adopt more policies than they abolish (see e.g., Adam et al., 2019; Hacker, 2004; Hinterleitner et al., 2023; Howlett & Rayner, 2013; Jakobsen & Mortensen, 2015; Kaufmann & van Witteloostuijn, 2018; Kay, 2007; Pierson, 2007; Steuerle & Kawai, 1996; Thelen, 2004).

However, the existing literature is less clear on *why* this policy growth is happening. There are at least four different main explanations for why modern democracies are characterised by incessant policy growth. The first explanation suggests that policy growth occurs because citizens in modern democracies want governments to address an ever-greater range of issues and problems. The second explanation suggests that policy growth results from interest group politics, with interest groups acting as strong policy demanders. The third explanation suggests that policy growth is an artifact of electoral competition, which induces politicians to engage in legislative activism and produce more policies than would actually be needed to address a given problem. The fourth explanation considers policy growth to be the consequence of institutional fragmentation, which forces politicians to agree on ‘messy’ and overly complex policy compromises to overcome veto points and to bridge political divides. While all four explanations have their merits, there is no study that assesses their relative importance and tests their predictions within a single empirical analysis and for a large number of cases over time.

This article seeks to address this lacuna by analyzing the drivers of policy growth in 21 OECD countries from 1976 to 2020 in the area of environmental policy. We present an event history analysis that assesses whether changes in public demands, the organisation of interest group politics, the intensity of electoral competition, or institutional fragmentation can better account for policy growth events in our country sample. This type of analysis allows us to identify each explanation’s likelihood of triggering policy growth. While *single* policy reforms could be easily explained by a focus on individual cases, the very concept of policy growth, i.e., the constant addition of new policies to existing policy stocks, requires that we adopt an aggregate perspective. We find that interest group politics have the strongest effect on policy growth. In comparison, the impact of public demands and institutional factors is less pronounced. The intensity of electoral competition, in turn, seems to have no substantive impact on policy growth.

These findings have important implications for the functioning of democracy in the long run. Research on the ‘policy state’ (also referred to as ‘activist state’ or ‘activist government’) has pointed to the political and institutional transformations associated with continued policy growth (e.g., Hacker & Pierson, 2014; Jenkins & Milkis, 2014; Mettler & SoRelle, 2014; Orren & Skowronek, 2017; Pierson & Skocpol, 2007). Many scholars consider policy growth to be an important marker of societal and democratic progress that indicates that citizens compel their governments to acknowledge and address an ever-greater range of issues and problems. However, scholars have also pointed to the negative repercussions of policy growth. By creating ever-more policy implementation tasks, policy growth threatens to overburden bureaucracies (Adam et al., 2019; Fernández-i-Marín et al., 2023b; Gratton et al., 2021; Limberg et al., 2021). Democratic governments that respond to an increasing range of public demands may therefore slowly entrap themselves in a situation where they make promises they cannot keep, as poorly implemented policies fail to make the promised impact (Fernández-i-Marín et al., 2023c).

By highlighting the reasons for this ‘predicament’ (Orren & Skowronek, 2017), our findings provide important insights into how democracies may cope with policy growth in the long run. If increased public demands were the primary driver of policy growth, democracies would face a simple choice between radically expanding administrative capacities (so that bureaucracies can cope with increased implementation tasks) or simply limiting their responsiveness to public demands – a development that would deprive them of one of their ‘unique selling propositions’ in the system competition between democratic and authoritarian regime forms (Milanovic, 2019). Since our analysis suggests that interest group politics and institutional fragmentation are equally important, if not more decisive, drivers of policy growth than public demands, democracies have at least *theoretically* the chance to reform themselves out of trouble by restructuring interest group politics and reducing institutional fragmentation. Overall, our findings provide important inputs for the debate about the future and sustainability of the democratic state.

The article proceeds as follows. *Section 2* presents four dominant theories of policy growth and fleshes out their empirical manifestations. *Section 3* explains how we measured the growth of sectoral policy portfolios (the dependent variable) and how we captured the dynamics expected to drive this growth (independent variables). *Section 4* walks readers through the various steps of the statistical analysis, and *Section 5* discusses the implications of our findings for long-running democratic developments.

## Theories of policy growth

Policy growth has been identified as a central feature of modern democracies. Both research that approximates the extent of policy growth by assessing the length of legal documents (e.g., Jakobsen & Mortensen, 2015; Jennings et al., 2005) and that assesses the number of policy instruments contained in legal documents (Adam et al., 2017) indicates a marked increase in rule growth, regardless of the country or policy sector under study (Adam et al., 2019; Hinterleitner et al., 2023).

An important and largely uncontested precondition for policy growth is that it is usually easier for politicians to simply add new policies to existing policy stocks than to replace policies that are flawed or have lost their purpose. As Bardach (1976, p. 123) already observed, while policy termination is often contemplated, ‘it is not often attempted and it rarely succeeds.’ Policy termination or ‘dismantling’ is unattractive for politicians because, over time, policies create layers of support that make their abolishment increasingly unattractive (Jordan et al., 2013; Pierson, 1994). Policies act as focal points for organised activity because they provide interest groups with resources and incentives to organise. The literature on policy termination is thus full of examples where termination attempts were unsuccessful (Geva-May, 2004). Hence, ‘new’ policies usually result in greater overall policy stocks over time, and research on the drivers of policy growth thus concentrates on the political dynamics that lead to the adoption of ever new policies.

Our study of the drivers of policy growth focuses on dynamics that can explain changes in *policy outputs*, i.e., changes in rules and regulations addressing concrete policy targets (e.g., the reduction of CO_2_ emissions from industrial plants) and policy instruments designed to reach these targets (e.g., emission standards, financial incentives, etc.). Policy growth hence primarily occurs through the continuous accumulation and expansion of governmental toolkits in different policy sectors. While policy growth can theoretically be studied by examining changes in public budgets (Jones et al., 2009), there may not always be a direct and simple relationship between budgets and policy stocks. For instance, changes in the level of welfare spending alone do not provide insight into the underlying policy portfolio’s configuration that led to those changes. It is even conceivable that a decrease in spending could be linked to an increase in the number of policies in the portfolio and vice versa (Adam et al., 2019). In the following, we hence take a closer look at theoretical accounts for the growth of sectoral policy outputs. We distinguish between four dynamics and flesh out their empirical manifestations.

### Explanation 1: policies accumulate because politicians respond to public demands

A first explanation suggests that policy growth is primarily due to democratic societies’ exposure to modernisation processes. Modern societies experience a steady stream of economic, demographic, technological, and societal changes and resulting challenges. While these changes have many indisputably positive effects such as greater affluence, health, comfort, and opportunities, they also come with an increased range of problems and threats. For example, while the invention of the automobile increased people’s mobility, it also raised the issue of road safety. While the rise of the internet increased people’s communication possibilities and provided them with dating platforms and shopping opportunities, it also came with the threat of cyberattacks and the poisoning of political communication.

There is ample evidence that citizens want governments to address these problems. Free and Cantril (1967, p. 37) first noticed the public’s ‘schizoid combination of operational liberalism with ideological conservatism.’ In other words, while many citizens report being generally against ‘big government,’ the picture is very different when citizens are asked whether the state should address *specific* issues such as the reduction of income differences or the provision of health care (Adam et al., 2019, pp. 34–35). Moreover, it seems as though citizens have become used to the fact that governments protect them from all kinds of harms, hazards, threats, and risks, ranging from disease outbreaks to industrial accidents to terrorist attacks to instances of consumer fraud (Ansell, 2019).

The primary way that governments address public demands and protect citizens from threats is through the production of public policies. Policies are governments’ No. 1 problem-solving tool because they allow them to deal ‘with issues and problems as they arise’ (Orren & Skowronek, 2017, p. 3). Policies’ flexibility and forward-looking nature are also the reasons why, historically, political actors developed strong incentives to use them to address the demands of their constituents. Theories of representative democracy thus consider policy adoption as a crucial ‘representation activity’ – which, in the long run, increases existing policy stocks. Politicians and parties compete for citizens’ votes by articulating their preferences and aggregating them in party programmes. Parties gain access to government power through electoral competition and subsequently transform citizens’ preferences into public policies (Caramani, 2017; Powell, 2004). For instance, the classical analysis by Higgs (1987) shows that ‘big government’ emerged from repeated US governmental responses to national emergencies such as the Great Depression, two World Wars, the Cold War, and various minor ‘crises’ (real or imagined). To address these emergencies, governments adopted a host of new federal programmes, activities, and functions. These changes had lasting effects, including a greater acceptance of a larger government, which persisted even after each crisis had subsided. The work by Jones et al. (2019) comes to a similar conclusion. As a result of public demands from broad social movements especially during the 1960s and 1970s, there was an immense broadening of the US government’s involvement into areas that had previously been off limits. This development of policy area expansion was then followed by a pattern of ‘thickening,’ entailing constant policy growth within established policy domains. In this logic, if public demands were to cause policy growth, *we would expect to primarily see policy growth in situations where issues are publicly salient*.

### Explanation 2: policy growth is the result of interest group politics

While Jones et al. (2019) discuss interest group politics mainly as a consequence of policy growth, many political scientists have argued and shown that interest groups have an important influence on public policy (e.g., Bawn et al., 2012; Gilens & Page, 2014; Hacker & Pierson, 2014). By their very nature, interest groups care strongly about specific issues. They are also usually much better organised and informed than ordinary citizens. Informational and organisational resources, in turn, allow them to survey the actions of politicians and parties closely, voice their demands in coherent and effective ways, and lure like-minded politicians through financial and/or organisational support (Hacker & Pierson, 2014; Rommetvedt et al., 2013). All these factors make it hard for politicians to ignore the demands of interest groups.

Yet, the extent to which demands from interest groups result in the adoption of new policies varies across countries and sectors and depends on the organisation of interest group politics. There are several reasons why interest groups’ impact on policy growth can be expected to be higher in corporatist than in pluralist arrangements. Corporatism is characterised by the integration of various societal interests in sectoral peak associations and multi-partite negotiations of policy options between these associations and the government. Corporatist arrangements thereby provide organised interests with privileged access to policy-makers (Ehrlich, 2011; Fernández-i-Marín, et al. 2023a). At the same time, governments benefit from the fact that they can deal with the already pre-coordinated positions of varying interest groups (Lehmbruch, 1987). These arrangements generally facilitate the transformation of interest group demands into policy adoption. Empirical evidence shows, for instance, that corporatist arrangements tend to generate more environmental policy innovations (Leyva-de la Hiz, 2019). In contrast, the relationship between interest groups and the government is less straightforward in pluralist systems where fragmented interest groups who compete for political access may mutually undermine and outbalance each other in their efforts to gain political influence. Hence, if the organisation of interest group politics were to drive policy growth, *we would primarily expect to see policy growth in settings where interest group representation is based on corporatist rather than pluralist arrangements.*

### Explanation 3: policies accumulate because electoral competition results in ‘too many’ policies

Policy growth need not necessarily result from societal demands but may also be a side effect of electoral competition. This ‘supply-centered’ explanation of policy growth usually starts from the assumption that politicians are self-interested and mainly motivated by career and (re-)election concerns (Downs, 1957; Mayhew, 1974). For politicians primarily interested in their performance in the next elections, policy adoption becomes a signal of political activism and commitment. The general expectation of so-called ‘electoral cycles’ in policy-making goes back to Nordhaus (1975), who argued that governments are likely to boost policy production prior to elections to maximise their chances of reelection. While this ‘strategic’ production of public policies has been identified as a more universal feature of democratic government, subsequent work has stressed that both the exact shape and strength of electoral cycles depend on many other factors, such as information asymmetries or institutional limitations (De Haan & Klomp, 2013; Dubois, 2016). Gratton et al. (2021) argue that the strategic considerations behind policy production are particularly pronounced in times of intense electoral competition and limited political time horizons. Under these conditions, so their argument goes, politicians have a particularly strong incentive to ‘overproduce’ policies so that voters view them as competent and skillful lawmakers. Moreover, the (higher) chance of losing the upcoming elections simultaneously reduces politicians’ risk of being confronted with the negative consequences of ill-conceived policies (Hinterleitner, 2020). Accordingly, if policy growth were driven by electoral competition, *we would primarily expect to see policy growth when electoral competition is intense*.

### Explanation 4: policies accumulate because of institutional fragmentation and the need for compromises

Finally, policy growth may be the result of the institutional make-up of the political system in which policy-making processes are embedded. In spite of the high variety of institutional arrangements across countries that include parliamentary, presidential, federal, and centralised systems, the existing literature points to some general institutional characteristics that either facilitate or constrain the adoption of new policies. Institutions have frequently been conceptualised as veto points that policy plans must pass before becoming reality (Tsebelis, 2002). While one could expect that by exacerbating policy adoption, veto points actually work against policy growth, a plurality of veto points may also have the exact opposite effect. As Teles (2013) has argued, veto points such as federalism or the committee structure in the US Congress can be conceived as ‘toll booths’ – with the toll taker demanding additional provisions or exceptions to policy proposals. This often results in cobbled-together policies that contain a multitude of provisions and components that are meant to appease opponents and secure legislative passage. Accordingly, if institutional fragmentation causes policy growth, *we would primarily expect to see policy growth in political contexts characterised by high institutional fragmentation.*

## Research design

We test the predictions of the four explanations against each other by analyzing environmental policy in 21 OECD countries over approximately four decades (1976–2020). The countries analyzed are Australia, Austria, Belgium, Canada, Denmark, Finland, France, Germany, Greece, Ireland, Italy, Japan, the Netherlands, New Zealand, Norway, Portugal, Spain, Sweden, Switzerland, the United Kingdom, and the United States of America. Thus, within the broader sample of developed countries, we employ a ‘diverse case’ selection strategy (Seawright & Gerring, 2008). A diverse case selection ensures a high degree of representativeness while allowing for the variation to be exploited in theoretically relevant variables for systematic comparison. While all these countries are advanced democracies, they differ substantially with regard to both the level of policy growth and the key explanatory variables (see Table 4 in the Online Appendix).

The measurement of policy growth requires an approach that goes beyond the analysis of individual policy changes and takes an aggregate perspective on policy developments at the level of policy domains or policy sectors. In this paper, we focus on environmental policy. Environmental policy is a relatively young policy area that has experienced strong policy growth over the last decades (Sommerer & Lim, 2016). Consequently, we can study the emergence of policy growth in this area from both the ‘infancy’ of a policy area and over a relatively long period of time. Moreover, environmental policy can be considered a rather ‘typical’ policy area in the context of our study. It is not only firmly embedded across the globe in national political systems and represented and institutionalised by ministries and parties (Biermann, 2021) but also constitutes no exception with regard to the overall trend of growing policy stocks that might bias our results. Rather pronounced increases in the size of sectoral policy stocks have also been reported for many other areas, including social policy (that displays a much longer history) or morality policy (where growing policy stocks coincide with an overall development towards more permissive policies) (Adam et al., 2019).

### Measuring policy growth

There are multiple approaches to measuring policy growth, including a focus on governmental spending (Pierson, 2007), on the number of lines, paragraphs, and words in legislation (see, e.g., Jennings et al., 2005; March et al., 2000; van Witteloostuijn & de Jong, 2010), and on the number of policy targets and policy instruments covered (Adam et al., 2019; Fernández-i-Marín et al., 2021). In this article, we focus on the number of policy targets and policy instruments. Focusing on changes in the size of sectoral policy portfolios (target-instrument combinations) overcomes the shortcomings of alternative approaches. First, governmental expenditure is definitely a good approximation for policy growth in areas in which more policies typically go hand in hand with more spending. This is usually the case in the area of social policy (Jensen, 2011). In areas in which governments primarily rely on regulatory approaches, however, a focus on governmental expenditure struggles to adequately capture changes in governmental intervention over time. Second, cross-country comparisons based on document amounts and lengths may be misleading given that countries substantially differ in their legal traditions and hence in the number (and length) of the laws adopted. For instance, Cooter and Ginsburg (2003) show that the exact same EU policy provision has resulted in very different statutes and judicial opinions length in the member states and that both aspects are associated with the overall lawyer population in a country. Meanwhile, looking at policy targets and instruments allows us to study the actual content of public policies and thus moves beyond an analysis of the formal features of legislation and associated problems with cross-country comparisons.

We identified the number of policy targets (*what exactly is being addressed?)* and instruments (*how is it addressed?*) in place based on a content analysis of laws and regulations carried out within the CONSENSUS and the ACCUPOL projects. Changes in policy targets and policy instruments were assessed by scrutinising all relevant national legislation that had been adopted throughout the observation period. We collected national legislation through national legal repositories and other legal databases such as ECOLEX. Additional checks on data reliability were carried out using legal commentaries and secondary literature. A detailed coding manual helped to systematically extract relevant information (policy targets and instruments) from the legal documents. We identified the 48 most addressed policy targets across three policy subfields that make up environmental policy: clean air, water conservation, and nature conservation policies. Moreover, we distinguished between 12 types of policy instruments (plus one residual category). These instruments cover ‘hard’ (obligatory standards, prohibitions, taxes, permits, etc.) and ‘soft’ (subsidies, public investments, information provision, voluntary instruments, etc.) forms of governmental intervention. In modern democracies, a range of different policy instruments typically address environmental policy targets (Gunningham & Sincalair, 1999). Section A of the Online Appendix lists all the identified and analyzed policy targets.

**Figure 1.**
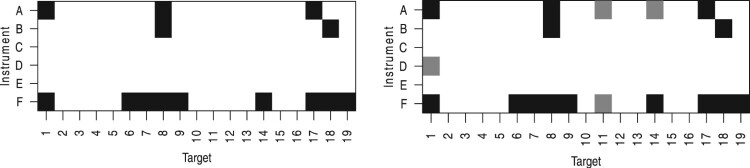
Example policy portfolios of a single country at two points in time.

Figure 1 illustrates our dependent variable. It shows two fictional policy portfolios that consist of policy targets (horizontal dimension) and policy instruments (vertical dimension). We speak of policy growth events whenever a new target-instrument combination is added to the policy portfolio. In the given illustration, the gray boxes represent the new target-instrument combinations added to the existing policy mix.

Empirically, the countries in our sample grew (on average) from covering 40 target-instrument-combinations in 1976 to 115 in 2020. This equals a 180 per cent increase in the policy portfolio size. At the end of our investigation period, the country with the smallest policy portfolio is Japan with 46 target-instrument-combinations covered. The county with the largest policy portfolio is France with 230 environmental policy measures in place. These descriptive insights suggest that (1) policy growth is (indeed) a major feature of modern democracies but that (2) we can also find substantial variation across different contexts. In the following, we introduce the operationalisation of the key variables expected to drive policy growth.

### Capturing public demands

Measuring public demand is a highly challenging endeavour. The literature proposes different approaches for measuring public demands (for an overview, see Oehl et al., 2017). The standard approach is to rely on public opinion data gathered through surveys (see e.g., Rasmussen et al., 2018; Wlezien & Soroka, 2016). The main advantage of survey data is that it *directly* measures people’s interest in and opinion on a given matter. The problem is, however, that surveys typically fail to provide data on many countries or consistent time series data for longer periods, or information on observations that date back in time. Given these challenges, we opt for a different approach that focuses on ‘party (system) salience’ and looks at the average mentions of certain issues in party manifestos (Dennison, 2019; Helbling & Tresch, 2011). This approach rests on the assumption that parties are responsive to the public when taking positions on a given policy issue. Public and party salience can diverge due to several factors. For instance, parties may occasionally misinterpret or underestimate the true concerns of citizens (Belchior et al., 2023). Moreover, parties might prioritise certain issues over others in view of their prevailing ideological orientations or in order to gain a competitive edge over their political opponents (Hillen, 2022).

Yet, our theoretical argument essentially refers to what parties consider to be publicly salient or want to make salient, as only in these cases can we realistically assume that public demands actually result in the delivery of public policies. So while our approach of measuring public demands certainly has its limits, we deem ‘party system salience’ to be an appropriate measure for capturing the impact of public demands on policy growth in the context of this study. We measure the average mentions of environmental issues (‘501, Environmental protection’) relative to the overall length (number of quasi-sentences) of party manifestos. This information is provided by the Manifesto Project Database (Volkens et al., 2021). In a second step, we take the mean value across all parties in a given country. By focusing on the average mentions of environmental policy matters across all party manifestos, we make sure not to confuse party system salience with the party position of the government.^1^ In other words, if environmental issues become more important to the broader public, the manifestos of political parties running for elections should also reflect this. Romeijn (2020) shows that there is a general link between public preferences and the positions of political parties but that this link weakens considerably once political parties are in government. By focusing on the average mentions of environmental matters across *all* party manifestos, we thus avoid potential problems of endogeneity, namely that the parties in power simply deliver what they have promised prior to the election – with or without responding to the people’s demands. The values between the elections are interpolated and smoothed (see Section E1 of the Online Appendix). We are fully aware that this salience-based approach only provides an indirect measure of public demands and primarily captures instances in which political parties have already somewhat reacted to public demands by incorporating these issues into their party manifestos. When presenting the empirical findings of our analysis, we further discuss the adequacy of this approach and provide additional support for its overall validity.

### Capturing the organisation of interest group politics

To assess the influence of interest group politics, we measure corporatism levels across countries. Although the concept was initially developed in the context of welfare state policy and industrial labour relations, it has also become a crucial explanatory variable in the context of environmental policy (Kronsell et al., 2018). Here, the central argument is that despite the traditionally strong dominance of *economic* interests in corporatist systems, a corporatist style of policy-making, characterised by the systematic inclusion of multiple interests and the tradition of achieving broad-based agreements, helps to ease conflicts and facilitate compromises (Crepaz, 1995; Jahn, 2000; Jahn, 2016a; Scruggs, 1999; Scruggs, 2001). These features make corporatist arrangements amenable to the production of environmental policy outputs (Skou Andersen, 2019).

While multiple datasets provide information on corporatist arrangements, most of them only cover a small set of countries or do not indicate change over time. Hicks and Kenworthy (1998) and Siaroff (1999) have created indices that measure corporatism at intervals, typically once per decade. However, this approach captures limited temporal variance, with changes occurring only every ten years. A particularly useful solution is offered by Jahn’s corporatism index (Jahn, 2016b), which covers a large number of countries and provides a time-variant measure of a country’s level of corporatism.

Jahn’s index covers both the structural and functional features of corporatist arrangements and the extent to which the economy is encompassed by the agreements made. By and large, the index aligns well with existing scholarly contributions on the topic. It must be noted, however, that due to the stronger consideration of temporal changes, Sweden, Switzerland, and the UK have a somewhat lower, while Ireland has a slightly higher corporatist score than what we would expect from other corporatist research.

### Capturing the intensity of electoral competition

We assess the intensity of electoral competition based on two interrelated factors. From the government’s perspective it is important to know whether (1) voters will change their vote from one party to the other and (2) whether these vote shifts will ultimately make a difference for the electoral outcome, i.e., the legislative seat share. While the first factor can be influenced by the government’s actions such as the policies it adopts or the announcements it makes, the latter is determined by a country’s electoral rules, institutions, and the geographic distribution of the electorate (Chen & Rodden, 2013). Kayser and Lindstädt (2015) combine these aspects in a single index of electoral competition to estimate the ‘perceived loss probability’ of the parties in government (which is readily available online).

### Capturing institutional fragmentation

To capture the degree of institutional fragmentation and the political need for compromises, we rely on work by Henisz (2000). Henisz provides a structurally-derived and internationally comparable measure of political constraints that focuses on two elements that have a strong bearing on the feasibility of policy change: ‘the number of independent veto points over policy outcomes and the distribution of preferences of the actors that inhabit them’ (Henisz 2000, 7). While the first element of this measurement is based on the number of veto points derived from the constitutional setup in a given polity, the second element captures whether the various actors controlling these veto points have the same or different policy preferences.

### Control variables

In addition to these variables of theoretical interest, we control for a range of other influences. First, we take into account that socio-economic and ecological developments may influence the likelihood of policy growth (Kristensen et al., 2023). Specifically, it may be the case that environmental problem pressure drives the adoption of additional policies. We control for environmental problem pressure by relying on data provided by Jahn (2016a), which tracks the development of key environmental pollutants such as SO_2_ or CO_2_ in our country sample. By using temporal lags, we ensure that shifts in environmental conditions *precede* and potentially drive changes in policy portfolios. This means if a correlation exists, we would first observe worsening environmental conditions, followed by subsequent alterations in the array of policies. Second, we include controls for the ideological orientation of ruling parties. Previous research has shown that in social policy, party ideology mainly impacts the type of policy growth rather than its extent (Fernández-i-Marín et al., 2023b). However, this dynamic might vary when it comes to environmental policy issues. Here, we look for the average position on the right-left dimension (Jahn, 2000; Neumayer, 2003) using the information provided by the Comparative Manifesto Project (Volkens et al., 2021). To determine the parties’ relative influence in government, we weigh the parties by their seat share. In addition, we control for the influence of international factors. For one, we code whether a country is a member of the European Union (EU). The EU has proven to be a prolific producer of public policies, particularly in the area of environmental and climate matters (Steinebach & Knill, 2017). Beyond EU effects, countries’ decisions to adopt new policies might result from international policy diffusion. Here, we expect that governments are more likely to follow one another when they are connected via trade ties. We hence control for these aspects by examining the share of goods being exported from one country to the other (Shipan & Volden, 2008). Lastly, economic factors may shape whether policy-makers propose additional policy measures. We control for this aspect using countries’ level of economic prosperity (GDP per capita) and level of public debt (indicated by the share of GDP). All our continuous variables are standardised to half a standard deviation so that we can directly compare their relative importance and compare continuous variables with binary ones (Gelman, 2008). Missing data are imputed using the time series average.

### Analytical model

We employ a frequentist event history analysis approach to estimate the likelihood of portfolio increases over time. Put simply, this analysis allows us to identify each explanatory variable’s likelihood of triggering a ‘policy growth event,’ i.e., the addition of one or more elements to the policy portfolio. The explanatory model consists of different levels and components. The outcome to explain is the extension (1) or not (0) of the policy portfolio for a given unit of analysis (*c*) during a given time period (*t*). Units of analysis are countries and time periods are weeks. Structuring our data this way allows us to take account of the cyclicality of policy-making. As demonstrated in *Section D* of the Online Appendix, the ‘baseline’ chance for policy growth is generally higher towards the end and towards the beginning of the legislative term – independent of the variables theorised above.

## Empirical Analysis

**Figure 2.**
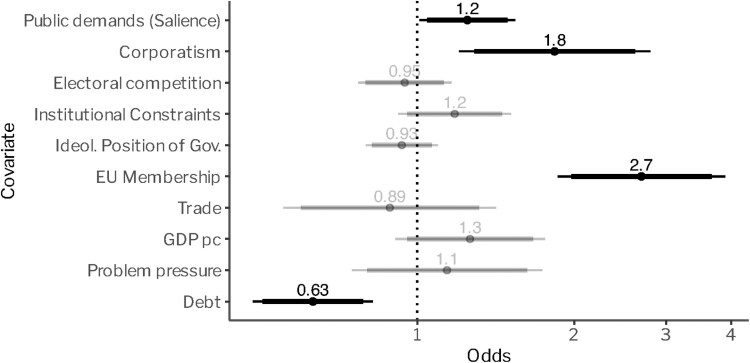
Determinants of environmental policy growth. Note: Our analysis covers 21 OECD countries over a period of 45 years (1976–2020). Note that all parameters are standardised to half a standard deviation and can therefore be roughly interpreted as the effect of an increase in one interquartile range; binary (EU) and continuous variables’ (all other) effects are directly comparable.

Figure 2 presents the results of our analysis. An exponentiated coefficient (odds) greater than ‘1’ indicates an increased chance of the occurrence of policy growth events. For a coefficient smaller than ‘1,’ the opposite is true. The analysis shows that governments are especially likely to accumulate policies when there are pronounced corporatist structures. The systematic inclusion of organised interests into the policy-making process and the practice of seeking broad-based agreements increase the chance of the adoption of an additional target-instrument combination by about 80 per cent.^2^

In addition to corporatism, greater public demands (salience) also generally increase the chance for policy growth, albeit to a lesser degree than corporatist arrangements. Governments are 20 per cent more likely to ‘accumulate’ policies when there is an overall greater demand for environmental protection. While we thus find support for the influence of organised interests and (to a lesser degree) public demands on policy growth, the intensity of electoral competition and institutional fragmentation play a negligible role.^3^

With respect to our control variables, the analysis confirms insights from previous research, namely that EU membership strongly drives policy growth in the area of environmental policy (↑ 170 per cent). Higher levels of debt, in turn, reduce the likelihood of portfolio expansion (↓ 87 per cent).^4^ Moreover, we find that environmental problem pressure is positively associated with policy growth. While pointing into the expected direction, this effect is not significant, however.

A possible reason for why we do not see significant effects of the intensity of electoral competition and institutional fragmentation could be that the aggregated perspective on policy growth obfuscates more nuanced influences of both factors on specific *types* of policy growth. To test for this possibility, we divided our dataset into two parts – one that captures the growth of ‘hard’ instruments (obligatory standards, prohibitions, taxes, permits, etc.) – and one that captures the growth of ‘soft’ instruments (information provision, voluntary instruments, public investments, etc.) (for a similar approach and distinction, see Schulze, 2021)*.* The underlying logic is that these two instrument types imply different levels of burden for the targeted actors in terms of the extent of required behavioural change and costs of compliance. Targeted actors might take this into account and thus only lobby for instruments that provide them with benefits rather than burdens (Schneider & Ingram, 1993). For instance, institutional fragmentation could lead to the growth of primarily soft instruments as the actors that hold important veto points may choose to add provisions to policy plans that directly benefit them or that leave them with considerable room to decide whether or not to utilise an instrument (such as public investments, subsidies, or voluntary instruments).

**Figure 3.**
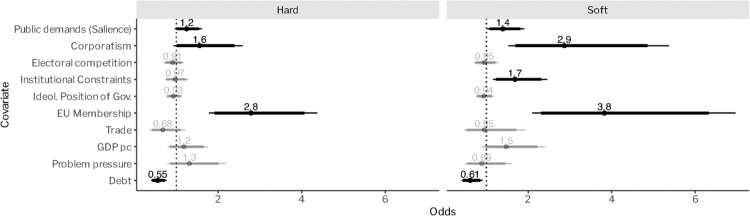
Determinants of environmental policy growth (disaggregated by instrument type). Note: Our analysis covers 21 OECD countries over a period of 45 years (1976–2020). Note that all parameters are standardised to half a standard deviation and can therefore be roughly interpreted as the effect of an increase in one interquartile range; binary (EU) and continuous variables’ (all other) effects are directly comparable.

Figure 3 replicates the analysis above but distinguishes between hard and soft policy instruments, thus revealing various insights (units of analysis are hard and soft policies clustered in countries). First, the separate analysis of hard and soft policy measures confirms the previous finding that the intensity of electoral competition makes no significant difference for policy growth. It thus seems that policy growth is not the result of politicians engaging in legislative activism. Second, we find that multiple ‘access points’ for organised interests are (again) a significant predictor of environmental policy growth – and that this is the case for both hard and soft policy instruments. Third, the analysis reveals that institutional fragmentation fuels the growth of soft (↑ 70 per cent) but *not* hard policy measures. This can be explained by the previously formulated expectation that the actors holding veto points primarily attach provisions to policy proposals that benefit them. In fact, the same could be expected for the influence of organised interests, and Figure 3 indeed shows that their influence is much stronger for soft (↑ 190 per cent) than for hard instruments (↑ 60 per cent). Finally, public demands have a largely similar effect on the growth of both hard (↑ 20 per cent) and soft instruments (↑ 40 per cent). This essentially implies that governments do not systematically prefer a certain instrument type for demonstrating their responsiveness to the citizenry.

In sum, our empirical analysis reveals that governments’ responses to public demands matter for policy growth but that they are overall less important compared with the organisation of interest group politics. Public demands and institutional fragmentation, in turn, seem to have a comparable influence on policy growth. While the effect of institutional fragmentation is stronger overall, its effect is limited to the adoption of soft policy instruments.

The important finding that public demands only play a secondary role in influencing environmental policy growth strongly depends on whether we adequately measure public demands. As discussed above, we assessed public demands by looking at the average mentions of environmental issues (salience) in all party manifestos in a given country. Moreover, we interpolated and smoothed the values between elections. Given this approach, one might criticise that our approach (1) only captures major demands that already made it onto the political agenda while (2) neglecting minor issues that occurred in-between elections.

To investigate the adequacy of our approach, we rely on innovative data provided by the Global Database on Events, Language, and Tone project (GDELT). The GDELT project monitors print, broadcast, and web news media across multiple countries to keep track of developments around the globe. We used the GDELT dataset to assess whether in the day-to-day news and events entries there were entries that include the text ‘Environment,’ ‘Global Warming’ or ‘Pollution.’ We then compared the number of entries with the total number of news and events to get a measure of the *relative* attention spent on environmental issues in a given country. This approach allows us to provide a daily assessment of the media attention spent on the environment for four years (2017–2021)^5^ and for all countries in our sample. While media attention is *not* exactly the same as public demands, previous research has demonstrated that media content is both a good reflection as well as an important driver of collective attitudes (see, e.g., Soroka et al., 2013).

**Figure 4.**
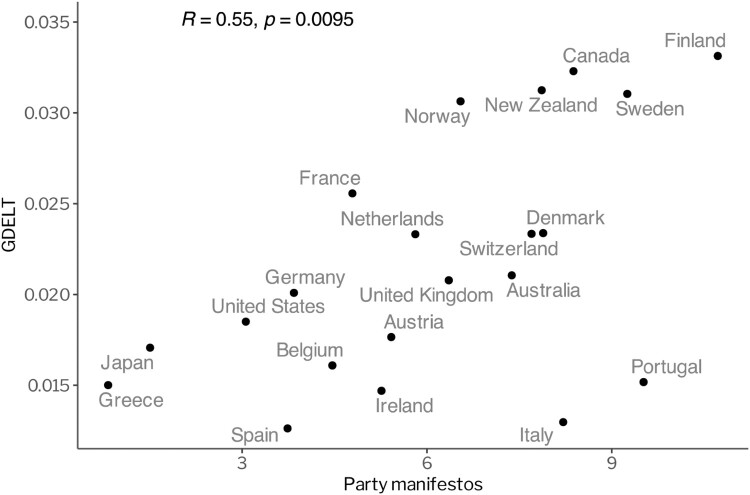
Correlation between the measures of salience for the environmental sector in media data and party manifestos, aggregated at the country level. Note: The figure compares environmental salience in media data and party manifestos for 21 OECD countries over a period of four years (2017–2021).

Figure 4 presents the country-level correlation between this measure and our measurement based on party manifestos. It shows that the different measures of salience are closely connected. The correlation between average issue attention and the salience in party manifestos during the observation period is 0.55 (99% confidence interval). The *temporal* correlation between daily relative attention and the smoothed party manifestos is slightly lower with a value of 0.35, but it is still highly significant (99% confidence interval). This finding lends considerable support to the overall reliability of our measurement of public demands. In fact, only in Portugal and Italy is salience in party manifestos largely detached from societal attention to environmental issues as measured by the GDELT data.^6^ We thus replicated our previous analysis (as shown in Figures 2 and 3) without these two countries. The central findings remain the same (see Figure 7 in the Online Appendix).

In addition to relying on GDELT data, an alternative approach would be to replicate our analysis with a ‘public salience’ measure using Eurobarometer data. Eurobarometer surveys are conducted regularly across Europe, asking citizens to indicate their top priority issues. In Figure 5 in the Online Appendix, we show that the Eurobarometer data displays comparable levels of correlation with our party manifestos measure as it is the case for the GDELT data. Yet, we also see that there is no clear directionality in the relationship in the sense that sometimes (changes in) the party system salience measure antecedes the public salience measure, and sometimes it is the other way around (see Figure 6 in the Online Appendix) – an observation that aligns with the previously made point that politicians not only respond to salient issues but also try to make certain things salient. While these findings support our measurement’s overall plausibility and validity, replicating our analysis with Eurobarometer data is hampered by the fact that it only partially captures our sample of countries under study. Seven of the 21 countries examined in this analysis (one third) are not included in the Eurobarometer survey. Additionally, conducting a meaningful analysis of just the subset of EU countries in our sample presents challenges. This difficulty arises from the observation that countries included and not included in the Eurobarometer survey exhibit significantly different values concerning the key explanatory variables of interest, as detailed in *Figure 1* in the Online Appendix*.*

## Conclusion

Policy growth has been identified as a pervasive and consequential development in modern democracies. This article examined potential drivers of this development – public demands, the organisation of interest group politics, the intensity of electoral competition, and institutional fragmentation – and assessed their relative importance within a single empirical analysis. By focusing on environmental policy growth in 21 OECD democracies over more than four decades, the analysis found that growing policy stocks are primarily driven by strong ties between organised interests and the government. Public demands and institutional fragmentation are also relevant but comparatively less important while the intensity of electoral competition has no influence on policy growth in our country sample.

Our article tested theories of policy growth in the area of environmental policy. The focus on environmental policy allowed us to examine policy growth from the emergence of a policy area and over a relatively long period of time. Moreover, environmental policy is in many ways a ‘typical’ policy area that has been strongly embedded in the institutional structures of modern democracies. However, some characteristics of environmental policy might separate it from other policy areas. One might argue that, in contrast to our assertions, environmental policy does not present a ‘typical’ but rather a ‘best case’ examination of our theoretical claims, given that it is a policy area that has undergone considerable and steady above-average growth over the past four decades. This, however, should affect our ‘baseline’ risk for policy growth and not so much the *within*-variation. However, it is important to acknowledge that the specific impacts of the factors under consideration might vary depending on the particular policy domains being scrutinised. For instance, one might expect that the reason behind our lack of a finding on a significant influence of the intensity of electoral competition is that environmental policies do not make a direct and immediate difference in citizens’ lives, and they therefore do not really lend themselves to engaging in legislative activism (as opposed to redistributing measures such as social policies). In other words, the strategic overproduction of public policies in response to fierce electoral competition might happen in areas other than environmental policy. In this context, one might also check for the differences within the area of environmental policy. For instance, it might be the case that in recent years, policy measures related to climate change have been particularly prone to policy growth compared to more traditional environmental regulation (for discussion, see Schulze, 2021). In light of these limitations, future studies interested in policy growth might take our findings as a starting point and test their validity in a broader range of policy areas.

These limitations notwithstanding, what do our findings imply for democracies’ long-term functioning and stability? We think that learning about the drivers of policy growth is a prerequisite for addressing the negative ‘side effects’ of this widespread phenomenon. As noted in the introduction, policy growth is in many ways associated with societal progress and modernisation. The adoption of new policies ideally means that governments address new societal and technological developments and manage the problems and challenges that are linked to them. However, and crucially, policy growth also threatens to gradually undermine the legitimacy of democratic systems because of ever-increasing implementation burdens and associated implementation deficits. If implementation bodies are increasingly overwhelmed by incessant policy growth, democracies are unlikely to actually deliver on their policy promises.

The finding that public demands are not the one and only driver of policy growth has far-reaching implications as it suggests that democracies do not necessarily create promises they cannot keep in the long term. If public demands were the main or exclusive driver of policy growth, then democracies would have to accept this reality and its implications for implementation ‘come hell or high water.’ On the contrary, our article, suggests that democracies are *not* necessarily doomed to constant policy growth and its problematic implications. Instead, it suggests that the problematic side effects of policy growth could be addressed or at least mitigated by restructuring interest group politics and reducing institutional fragmentation in policy-making processes.

## Supplementary Material

Supplemental Material

## Data Availability

All data and replication files is accessible via the following link: http://xavier-fim.net/articles/jepp-2024.

## Notes

1 In calculating average changes in party system salience, we do not weight the different parties in view of their vote share at previous elections. Political parties can be assumed to develop their manifestos to maximise their vote share in future elections, hence addressing the whole electorate. While different degrees of party responsiveness to certain issues might affect electoral success, our interest is to broadly capture changes in the average salience political parties attach to certain issues. In addition, we separately control for the government’s ideological position.

2 One might challenge this finding by arguing that it is not so much the system of interest intermediation that matters for policy growth but simply the number of activist groups (as emphasised by Jones et al., 2019). In the Online Appendix (Figure 9) we therefore provide an additional analysis showing that our findings hold even when controlling for the number of environmental NGOs (per capita) in a country.

3 We also tested for the possibility that electoral competition and institutional fragmentation only exert an *indirect* effect on policy growth by strengthening or weakening the effect of public demands. In Figure 5 in Section F of the Online Appendix, we show however that electoral competition and institutional fragmentation have *no* moderating effect on the relationship between public demands and policy growth.

4 To compare positive and negative odds, negative odds must be exponentiated.

5 A more comprehensive dataset covering a longer period of time is not yet published.

6 When dropping the cases of Italy and Portugal, the correlation between average issue attention and the salience in party manifestos increases to 0.79.
