# Supplementary material for: Testing theories of policy growth: public demands, interest group politics, electoral competition, and institutional fragmentation
Source: J Eur Public Policy. 2024 Feb 27;32(3):784–809. doi: 10.1080/13501763.2024.2317358 (PMC11835310; doi:10.1080/13501763.2024.2317358)
Supplement: Supplemental Material [file RJPP_A_2317358_SM5045.pdf]

Appendix for "Testing Theories of Policy Growth: Public Demands, Interest Groups, Electoral Competition, and Institutional Fragmentation"

Supplemental Online material

Abstract

Technical documentation for "Testing Theories of Policy Growth: Public Demands, Interest Groups, Electoral Competition, and Institutional Fragmentation".

Contents

A Coding manual (excerpt) . . . 2
A.1 Basic Coding Procedure and Main Concepts . . . 2
A.2 Coding Categories . . . 2
A.3 Coding Category 1: Policy Targets . . . 2
A.4 Coding Category 2: Policy instruments . . . 5

B Data description: Policy growth . . . 7

C Data description: explanatory variables . . . 8

D Temporal dynamic into legislature . . . 9

E Salience . . . 10
E.1 Comparative Party Manifestos (CPM) interpolation . . . 10
E.2 Comparison of sources . . . 11
E.3 Alignment . . . 12

F Robustness . . . 13

A Coding manual (excerpt)

A.1 Basic Coding Procedure and Main Concepts

At the most basic level, the coders have to identify single events of policy change in the collected legal documents and, for each single event, assess the direction of change, i.e., whether the event of policy change represents the introduction or abolishment of a given target-instrument-combination.

To come into consideration, a policy change has to meet the following requirements in form and content. Formally, a relevant policy change is any measure or provision in the collected legislation (and where necessary respective administrative circulars specifying these rules) that

- was published during the observation period, which starts on **January 1, 1976**, and ends on **March 31st, 2021**
- was adopted at the **national level**

The second point clearly excludes measures by sub-national jurisdictions such as regional or local bodies, even if the latter are state-like entities with far-reaching competencies as in federal states.

A.2 Coding Categories

The method used to assess and code policy change, is intended to be universally applicable, i.e. over a wide range of countries, irrespective of differing legal and administrative traditions. Thus, the coding rules comprise two invariant general categories. These are policy targets and policy instruments.

By means of these two categories, we seek to measure developments over time in a nuanced manner. Moreover, in order to assess whether a change represents the introduction or abolishment, we are interested in policy change relative to the previous state. Thus, as will be explained in more detail in this section, relative changes to the previous targets and instruments need to be coded. We are interested in the introduction and abolishment of (new) policy target (guiding question: *what is adressed?*), of policy instruments (*how is something addressed?*).

Recalling the observation period (January 1st, 1976 to March 31st, 2021), this stated focus on change has one important implication: Although the relevant information for deciding whether a legal act falls into the observation period is the date of publication, it might be the case that coders need to consult legislation originating from some year before 1980 in order to reconstruct the occurrence and the direction of change. For instance, if a law adopted in 2008 changes a law enacted in 1973, the latter legislation has to be considered in order to make a statement about the direction and nature of change taking place through the 2008 legislation.

A.3 Coding Category 1: Policy Targets

The first and most general coding category is policy targets. For analytical reasons, we use a very narrow conception of policy targets. By policy targets, we mean a very specific activity within a subarea of a policy field guided by the question: who or what is addressed? More specifically, a policy target is subject to state activities in order to achieve a political objective within a specific area. The tables below contain the policy targets this project is exclusively interested in. Thus, when screening the legislative acts, please identify the presence and/or abolishment of any policy targets from these lists and indicate these events of policy change as either introduction or termination.

One single target has to be coded only once per legislative act – it must not be coded multiple times. Any instrument concerning this specific target will be attributed to the one single target. If a policy target from the list is introduced for the first time, i.e. subject to governmental action for the first time, this particular event must be coded as policy introduction. If, by contrast, a policy target from the list is abolished, i.e. is not subject to governmental action anymore, this particular event must be coded as policy termination.

Please note that the termination of a target entails the termination of all attached instruments, which have to be coded separately. The same is true when a target is addressed for the first time.

Clean Air Policy

1. Air quality standards for nitrogen oxides (NOx)
2. Air quality standards for sulphur dioxide (SO_2)
3. Air quality standard for carbon monoxide (CO)
4. Air quality standard for particulate matter
5. Air quality standard for ozone (O_3)
6. Air quality standard for lead
7. Nitrogen oxide (NOx) emissions from large combustion plants using coal
8. Nitrogen oxide (NOx) emissions from passenger vehicles using unleaded gasoline
9. Nitrogen oxide (NOx) emissions from heavy duty vehicles using diesel
10. Sulphur dioxide (SO_2) emissions from large combustion plants using coal
11. Sulphur dioxide (SO_2) emissions from passenger vehicles using unleaded gasoline
12. Sulphur dioxide (SO_2) emissions from heavy duty vehicles using diesel
13. Carbon dioxide (CO_2) emissions from large combustion plants using coal
14. Carbon dioxide (CO_2) emissions from passenger vehicles using unleaded gasoline
15. Carbon monoxide (CO) emissions from large combustion using coal
16. Carbon monoxide (CO) emissions from passenger vehicles using unleaded gasoline
17. Particulate matter emissions from large combustion plants using coal
18. Arsenic emissions from stationary sources
19. Maximum permissible limit for the lead content of gasoline
20. Maximum permissible limit for the sulphur content of diesel
21. Carbon dioxide (CO_2) emissions from aviation activities
22. Maximum permissible limit for the sulphur content of petrol (gasoline, benzine, fuel)

Water Protection Policy

1. Lead in continental surfaces water (i.e. waters that flow or which are stored on the surface, and include natural water channels like rivers, surface runoff, streams, lakes and others)
2. Copper in continental surfaces water
3. Nitrate (NO_3^-) in continental surfaces water
4. Phosphates in continental surfaces water
5. Zinc in continental surfaces water
6. Oils in continental surfaces water
7. Pesticides (fungicides, herbicides, insecticides, exempt DDT) in continental surfaces water

8. DDT (Dichloro-Diphenyl-Trichloroethane) in continental surfaces water
9. Phenols (as total C) in continental surfaces water
10. BOD (Biochemical Oxygen Demand) of continental surfaces water
11. Lead from industrial discharges into continental surfaces water
12. Copper from industrial discharges into continental surfaces water
13. Nitrate (NO_3^-) from industrial discharges into continental surfaces water
14. Phosphates from industrial discharges into continental surfaces water
15. Chloride (Cl^-) from industrial discharges into continental surfaces water
16. Sulphates from industrial discharges into continental surfaces water
17. Iron from industrial discharges into continental surfaces water
18. Zinc from industrial discharges into continental surfaces water
19. Oils and greases from industrial discharges into continental surfaces water
20. Pesticides and herbicides from industrial discharges into continental surfaces water
21. Phenols (as total C) from industrial discharges into continental surfaces water
22. Coliform bacteria from industrial discharges into continental surfaces water
23. BOD (Biochemical Oxygen Demand) from industrial discharges into continental surfaces water
24. COD (Chemical Oxygen Demand) from industrial discharges into continental surfaces water

Conservation Policy

1. Native Forests
2. Nature protection areas and reserves
3. Import and export of endangered species
4. Import and export of endangered plants

A.4 Coding Category 2: Policy instruments

We define a policy instrument as a tool or means adopted to achieve the underlying political objective of the selected environmental policy target. A policy instrument thus describes the type of governmental action adopted for a given policy target. A policy instrument is intended to have a regulating and/or guiding effect on people's actions. The tables below contain all potential policy instruments for environmental policy. For each policy targets, if addressed, there is at least one policy instrument defined as a tool to achieve the underlying political objective. Yet, any policy target may be addressed by means of various policy instruments. For each addressed policy target, the coders are asked to identify all instruments. Please note that a given policy instrument belongs to one type/group only.

The following table is exhaustive, containing the most common environmental policy instruments.

Instrument	Description	Example
Obligatory standard	A legally enforceable numerical standard, typically involving a measurement unit, e.g. mg/l	Limit value for lead emissions in surface water, e.g. 50 mg/l
Prohibition / ban	A total or partial prohibition/ban on certain emissions, activities, products etc.	Ban on importation of products containing flurochlorocarbons
Technological prescription	A measure prescribing the use of a specific technology or process	Installations have to be operated in accordance with the principle of 'best available techniques' (BAT)
Tax / levy	A tax or levy for a polluting product or activity	Tolls and road user charges for trucks depending on the emission class

Subsidy / tax reduction	A measure by which the state grants a financial advantage to a certain product or activity	Tax reduction for vehicles in series production complying with a regulation
Liability scheme	A measure that allocates the costs of environmental damage to those who have caused the damage	Establishment of an emission trading system
Planning instrument	A measure defining areas or times that deserve particular protection	Action plans indicating the measures to be taken during times when there is a risk of the limit being exceeded
Public investment	A specific public investment	Programs given financial support for the retrofitting of in-use vehicles and for scrapping old vehicles
Data collection / monitoring programmes	A specific programme for collecting data	Establishment of measuring stations designed to supply the data necessary for the application of a certain regulation
Voluntary measures	Voluntary agreements or commitments between the state and private actors or by private actors alone	Manufacturers can apply for the CO2 savings achieved as a result of eco-innovation (if approved can used to contribute to manufacturer's specific emissions target)
Information-based instrument	Information provided by the state or the polluters indicating the environmental externalities of a certain product or activity	Label on fuel economy and CO2 emissions of a vehicle displayed at the point of sale.
Other	Any instrument that cannot be assigned to the other categories	(…)

B Data description: Policy growth

Table 3: Descriptive summary of policy growth.

Country	Policy Growth	
	% Time	Over time
France	2.46	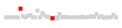
Austria	2.294	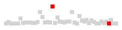
Denmark	2.21	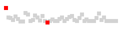
Belgium	2.168	
United Kingdom	2.043	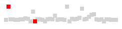
Italy	2.002	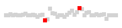
Netherlands	2.002	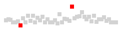
Greece	1.96	
Portugal	1.835	
Ireland	1.793	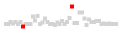
Germany	1.668	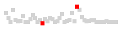
Sweden	1.626	
Spain	1.543	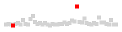
Finland	1.376	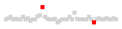
Switzerland	1.084	
United States	1.084	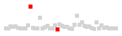
Canada	1.001	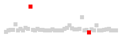
Norway	1.001	
Australia	0.6672	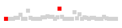
New Zealand	0.5421	
Japan	0.2502	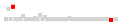

C Data description: explanatory variables

Table 4: Summary statistics. Explanatory variables.

Variable	N	Mean	Std. Dev.	Min	Pctl. 25	Pctl. 75	Max
Public demands (Salience)	50358	5.9	3.3	0.086	3.7	7.9	18
Corporatism	50358	0.084	0.68	-1.1	-0.42	0.6	1.5
Electoral competition	44902	0.25	0.22	0	0.037	0.45	0.75
Institutional Constraints	50358	0.47	0.093	0	0.41	0.53	0.72
Ideology, average	47852	5.6	1.4	1.8	4.4	6.7	8.7
EU Membership	50358	0.34	0.47	0	0	1	1
Trade	50358	69	35	16	45	83	252
Debt	50358	64	38	2.3	40	81	249
GDP pc	50358	29188	18734	2173	13663	41575	102913

Figure 1 helps comparing the range of ”Corporatism” when data for Eurobarometer’s salience is available or not.

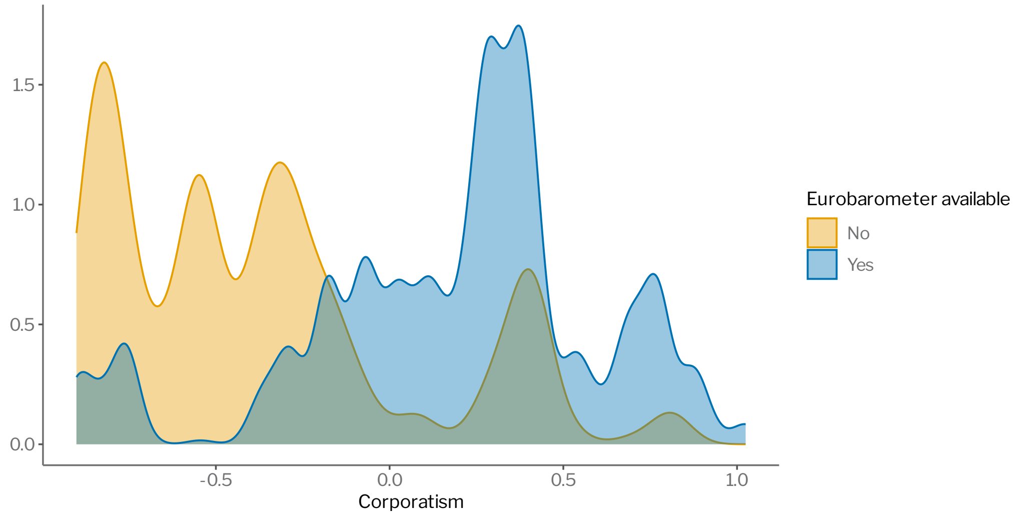

Figure 1: Distribution of Corporatism, with color indicating whether Eurobarometer data is available or not.

D Temporal dynamic into legislature

This section includes the baseline hazard of policy growth as the legislature moves on. This has been obtained by the splines of the generalized additive model that smooth the effect of the legislative period into the likelihood of portfolio growth.

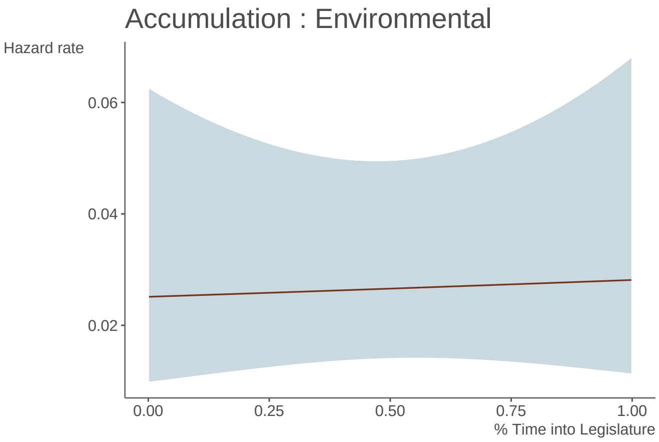

Figure 2: Baseline hazard of policy growth at different times into the legislature. Reference model.

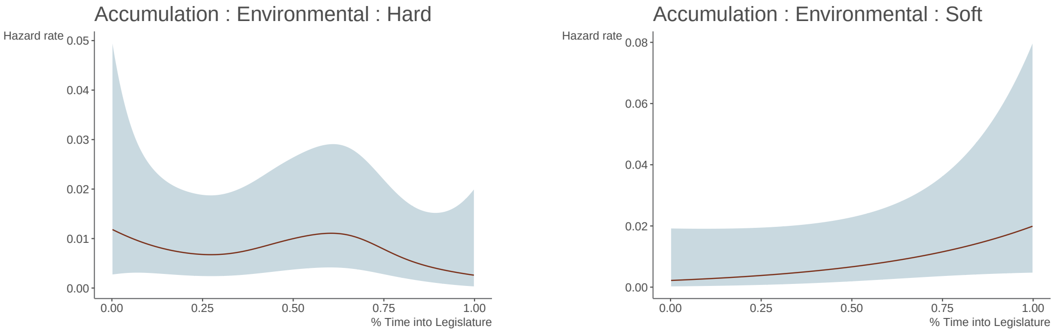

Figure 3: Baseline hazard of policy growth at different times into the legislature. Models with hard and soft instrument types.

E Salience

E.1 Comparative Party Manifestos (CPM) interpolation

Figure 4 shows the dates where data from party manifestos is available, along with the interpolated time series.

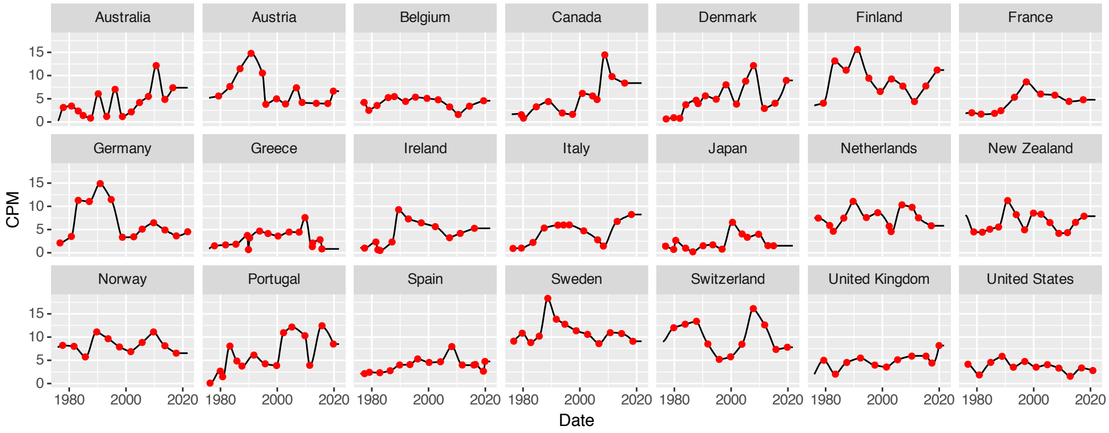

Figure 4: Interpolated time series of salience as measured by the comparative party manifesto data, using the dates of the elections and their respective manifestos (red).

E.2 Comparison of sources

Figure 5 shows the correlation between Salience, as measured by party manifestos (reference), through media attention (GDELT) or through public opinion (Eurobarometer).

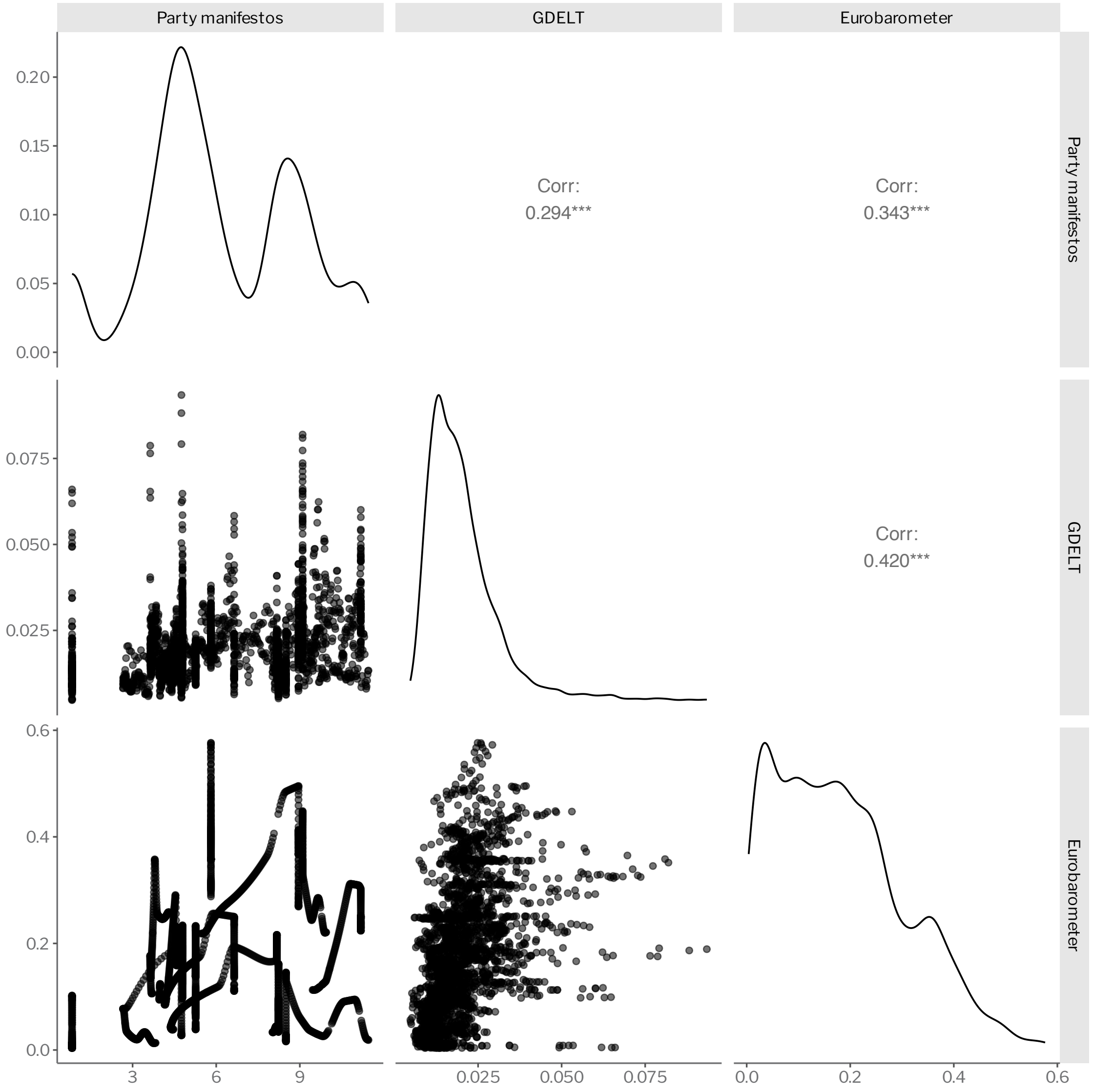

Figure 5: Correlation between salience measures.

E.3 Alignment

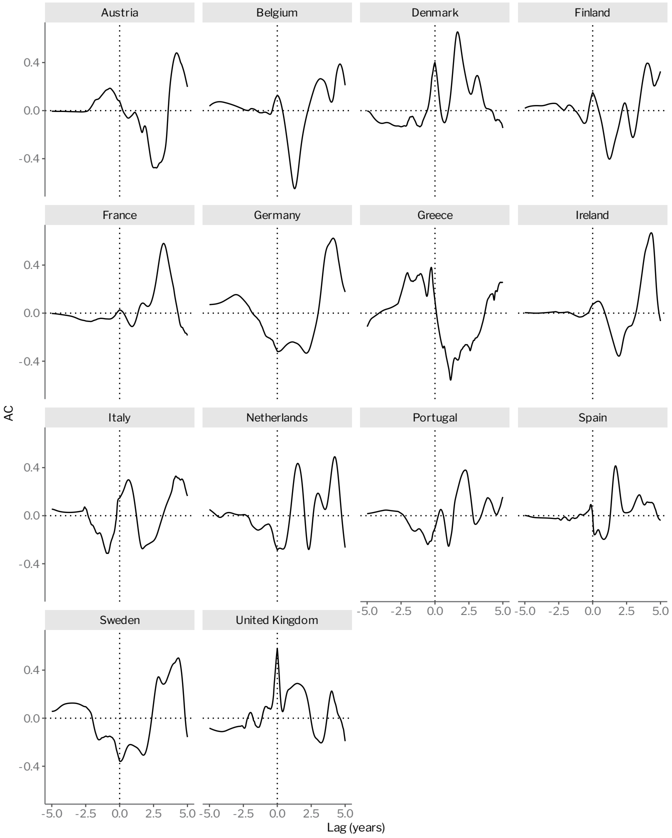

Figure 6: Time-lagged cross-correlations between Salience measured in Eurobarometer or Party Manifestos. When the lag is negative, salience changes in the Eurobarometer lead changes in Party Manifestos. When the lag is positive, salience changes in Party Manifestos lead changes in the Eurobarometer.

F Robustness

Figure 7 compares the reference model with one that does not include Portugal nor Italy.

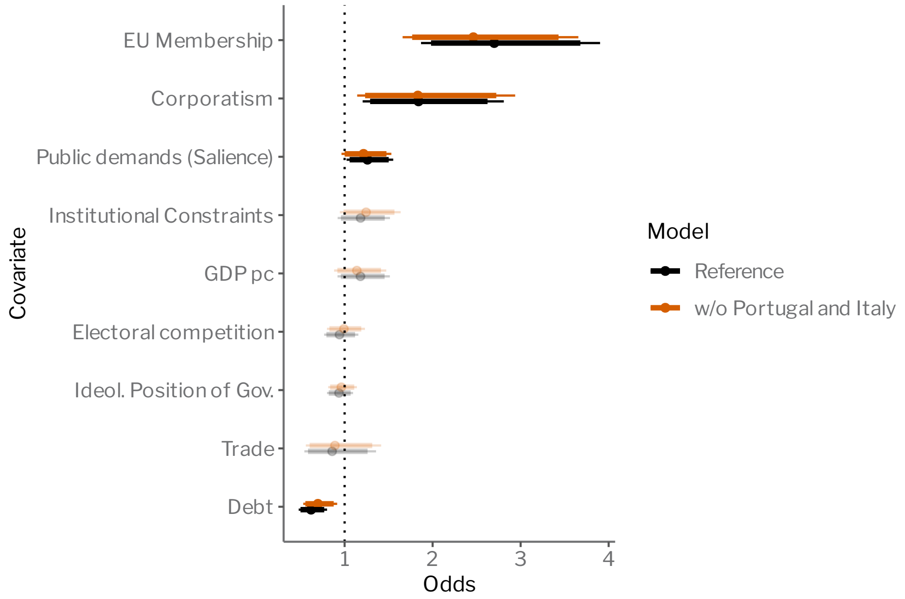

Figure 7: Model comparison between the reference and one without Portugal and Italy.

Figure 8 shows the results of a model specification with two added interactions involving salience: electoral competition and political constraints.

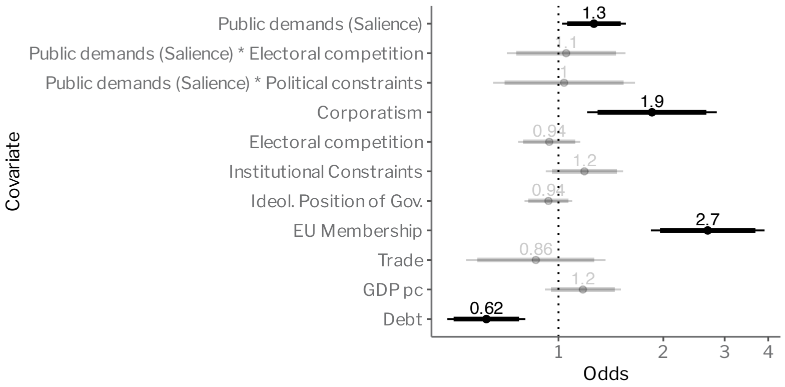

Figure 8: Model results for a specification where salience is interacted with electoral competition and political constraints.

Figure 9 compares the reference model with one that contains a control by Environmental NGOs.

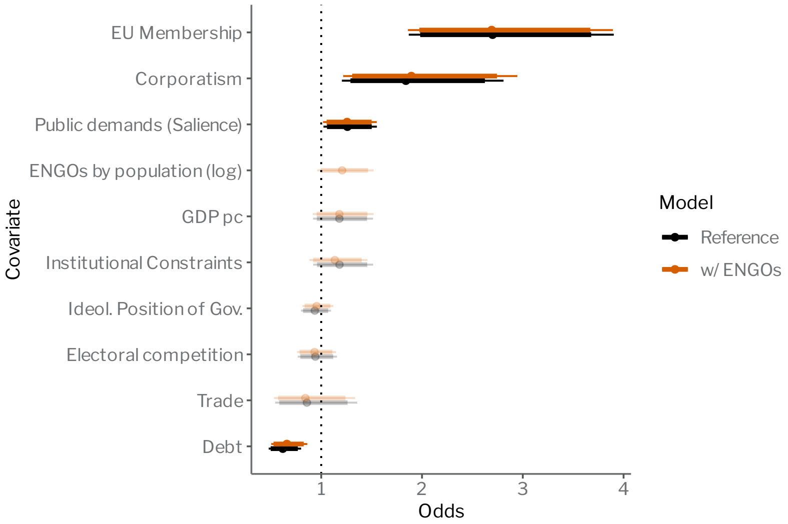

Figure 9: Model results for a specification containing a control by Environmental NGOs.
